# Asthma is Underdiagnosed and Often Uncontrolled in Preoperative Patients With Chronic Rhinosinusitis With Nasal Polyps

**DOI:** 10.1111/cea.14548

**Published:** 2024-07-25

**Authors:** Emma Genberg, Paula Kauppi, Johanna Sahlman, Anu Laulajainen‐Hongisto, Markus Lilja, Sari Hammarén‐Malmi, Lena Hafrén, Antti Mäkitie, Seija Vento, Sanna Toppila‐Salmi, Paula Virkkula

**Affiliations:** ^1^ Respiratory Diseases and Allergology University of Helsinki and Helsinki University Hospital Helsinki Finland; ^2^ Department of Otorhinolaryngology University of Eastern Finland and Kuopio University Hospital Kuopio Finland; ^3^ Department of Otorhinolaryngology, Head and Neck Surgery University of Helsinki and Helsinki University Hospital Helsinki Finland; ^4^ Department of Otorhinolaryngology University of Eastern Finland Joensuu and Kuopio Kuopio Finland; ^5^ Wellbeing Services County of Pohjois‐Savo Kuopio Finland; ^6^ Inflammation Center, Department of Allergology University of Helsinki and Helsinki University Hospital Helsinki Finland

**Keywords:** asthma, asthma control, chronic rhinosinusitis with nasal polyps, comorbidity, endoscopic sinus surgery

**Summary**

- Asthma prevalence was high preoperatively in patients with uncontrolled chronic rhinosinusitis with nasal polyps.
- Half of these patients undergoing endoscopic sinus surgery might have undiagnosed or uncontrolled asthma preoperatively.

To the Editor

Most patients with chronic rhinosinusitis with nasal polyps (CRSwNP) have type 2 inflammation, as do patients with eosinophilic asthma [1, 2, 3]. Asthma often accompanies CRSwNP [4, 5], and CRSwNP with comorbid asthma is associated with more severe sinonasal disease [5, 6, 7]. Furthermore, asthma is more prone to exacerbations when comorbid CRSwNP is present [5, 8]. The aim of this study was to describe the prevalence of asthma and evaluate asthma control in patients with uncontrolled CRSwNP undergoing endoscopic sinus surgery (ESS) at a tertiary centre. We also aimed to identify factors associated with asthma in this population.

This cross‐sectional study was conducted as part of a prospective controlled multicentre trial (AirGOs Operative) investigating the benefits of complete and limited ESS in patients with uncontrolled CRSwNP. Patients suffering from uncontrolled CRSwNP, a Sinonasal Outcome Test‐22 (SNOT‐22) score ≥30, a nasal polyp (NP) score ≥4, a Lund–Mackay (LM) score ≥14 and at least one of the following: ≥1 previous surgery for CRSwNP, ≥1 oral corticosteroid course or ≥3 antibiotic courses during the past 2 years were included in this study.

In addition to basic characteristics and comorbidities, the data in this study comprised preoperative nasoendoscopy, NP and LM scores, exhaled nitric oxide (eNO), blood eosinophil count (B‐eos), serum immunoglobulin E concentration (S‐IgE), serum allergen‐specific IgE measurements, Sniffin’ Sticks Screening 12 Test (SST‐12) and Health‐Related Quality of Life (HRQoL) of the upper and lower airways (SNOT‐22, Asthma Control Test [ACT] and 15D).

Asthma was assessed and diagnosed systematically. Previous asthma diagnosis was based on medical record information for physician‐diagnosed asthma. The prevalence of previous physician‐diagnosed asthma (AB), asthma diagnosed in the preoperative tests (preAB), and no asthma were calculated. All the patients underwent spirometry and a bronchodilator test and peak expiratory flow (PEF) recording preoperatively [9]. Methacholine challenge test was performed for patients with asthma symptoms (prolonged or night‐time cough, wheezing or shortness of breath with or without auscultatory wheezing) but without AB and with undiagnostic results in spirometry and PEF recording. In spirometry, improvement with a bronchodilator in forced expiratory volume in the first second (FEV1) or forced vital capacity of at least 12% and 200 mL was considered diagnostic for asthma [9]. In PEF recording, diurnal variability (≥20% and 60 L/min at least three times) or bronchodilator responsiveness (≥15% and 60 L/min at least three times) was considered diagnostic for asthma. In the methacholine challenge test, moderate or severe hyperreactivity (a cumulative dose of ≤600 μg inhaled methacholine causes a 20% reduction in FEV1) was considered diagnostic for asthma. Uncontrolled asthma in AB and preAB patients was defined as one or more of the following: ACT score <20 [9] or significant reversible airflow obstruction in PEF recording or spirometry.

Statistical analyses were performed with the chi‐squared test, permutation test, Kruskall–Wallis test, analysis of variance, *t*‐test, Mann–Whitney *U*‐test and logistic regression analysis for odds ratio.

Of the 87 patients with uncontrolled CRSwNP, 69 (79%) suffered from asthma preoperatively. Fifteen patients (17%) had previously undiagnosed asthma (preAB). Eighty per cent of the patients had severely decreased HRQoL, with SNOT‐22 ≥ 40. There were no differences between the AB, preAB and no‐asthma groups in sex, body mass index (BMI), mean age, prevalence of high scores in questions 6 and 14 evaluating cough and night awakening in SNOT‐22, ASA intolerance or NP, LM or SST‐12 scores. The prevalence of allergic rhinitis was more common in the AB and preAB groups (*n* = 28, 52%, and *n* = 9, 60%, respectively) than in the no‐asthma group (*n* = 3, 17%) (*p* = 0.017). B‐eos was elevated in all the groups (mean 0.54 E9/L, SD 0.41, range 0–2.84 in the whole study population) but did not differ between the groups (Table 1). In patients with AB, eNO was higher than in those with no asthma (*p* = 0.041), but the relationship between asthma (preAB or AB) and eNO was not significant after adjusting for smoking and allergic rhinitis (*p* = 0.11). Allergic rhinitis had a relationship with asthma (AB or preAB) in multivariate analysis (odds ratio 5.41, 95% CI 1.24–23.61) when sex, age, BMI, smoking status and ASA intolerance were included. There was a weak correlation between the SNOT‐22 and ACT scores (−0.23 [95% CI −0.42 to −0.01]).

**TABLE 1:** Characteristics of patients with uncontrolled chronic rhinosinusitis with nasal polyps (CRSwNP) undergoing endoscopic sinus surgery, with or without comorbid asthma.

	Asthma			*p* value (post hoc)^a^
	No asthma *N* = 18	Preoperatively diagnosed asthma (preAB) *N* = 15	Previously diagnosed asthma (AB) *N* = 54	
Male, *n* (%)	13 (72)	10 (67)	26 (48)	0.14
Age, years, mean (SD)	47 (11)	46 (11)	48 (11)	0.89
BMI, mean (SD)	26.6 (5.3)	30.4 (5.2)	27.4 (4.6)	0.058
Sleep apnea, *n* (%)	0 (0)	2 (13)	6 (11)	0.38
Smoking, *n* (%)	—	—	—	0.61
Never	5 (28)	6 (40)	22 (41)	—
Ex	10 (56)	6 (40)	23 (43)	—
Occasional	1 (6)	1 (7)	7 (13)	—
Current	2 (11)	2 (13)	2 (4)	—
Elevated serum specific IgE against aeroallergen, *n* (%)	7 (41)	10 (67)	28 (56)	0.34
15D score, mean (SD)	0.892 (0.065)	0.850 (0.090)	0.881 (0.080)	0.28
ACT total score < 20, *n* (%)	3 (17)	9 (64)	22 (42)	0.021 (no asthma/preAB)
Exhaled nitric oxide, ppb, mean (SD)	25 (14)	42 (51)	44 (46)	0.041 (no asthma/AB)
ASA intolerance^b^, *n* (%)	7 (39)	7 (47)	29 (54)	0.57
Allergic rhinitis^c^, *n* (%)	3 (17)	9 (60)	28 (52)	0.017 (no asthma/preAB, no asthma/AB)
Polyp score total, median (IQR)	6 (6.7)	6 (4.7)	6 (4.7)	0.44
LM‐score total, mean (SD)	19.6 (2.9)	18.3 (3.5)	19.6 (2.9)	0.33
Sniffin’ Sticks Screening 12 Test^d^ score 0–6 (anosmia), better nostril, *n* (%)	15 (88)	9 (60)	39 (78)	0.20
SNOT‐22 Q6^e^ score 4–5, *n* (%)	1 (6)	3 (20)	5 (9)	0.42
SNOT‐22 Q14^e^ score 4–5, *n* (%)	9 (50)	7 (47)	20 (37)	0.56
SNOT‐22 total score, mean (SD)	53 (15)	59 (16)	53 (15)	0.42
Tissue eosinophils,^f^ %, median (IQR)	50.0 (25.0)	30.0 (36.0)	50.0 (40.0)	0.12

Abbreviations: ACT, Asthma Control Test; BMI, body mass index; IQR, interquartile range; LM, Lund–Mackay; SNOT‐22, Sinonasal Outcome Test‐22; SD, standard deviation.

^a^ Sidak's multiple comparison procedure was used to correct significance levels for post hoc testing (*p* < 0.05).

^b^ ASA intolerance was defined as a history of respiratory symptoms within 2 h after nonsteroidal anti‐inflammatory drug use or with a positive challenge test against ASA.

^c^ Allergic rhinitis was defined as physician‐diagnosed allergic rhinitis or allergic rhinitis symptoms and positive IgE against common aeroallergens.

^d^ Scores from 0 to 6 indicate anosmia, and scores from 7 to 10 indicate hyposmia.

^e^ Questions 6 (cough) and 14 (waking up at night) in the SNOT‐22 questionnaire.

^f^ From nasal samples preoperatively, after a 5 to 7 day pause in corticosteroids.

Asthma was uncontrolled preoperatively in 40 (58%) patients suffering from asthma. Sex, age, BMI, smoking status, ASA intolerance, allergic rhinitis, B‐eos, NP score, LM score, anosmia and eNO did not differ between the controlled and uncontrolled asthma groups.

The small sample size was a limitation of this study, and the results should be verified with studies in other populations and with larger sample sizes. These results are likely to be applicable to patients with the clinical assessment criteria used and an indication for surgery. Most patients in this study had severely decreased HRQoL, with a SNOT‐22 score of at least 40. The strengths of this study were its systematic evaluation of HRQoL with the SNOT‐22, ACT and 15D questionnaires and its concurrent examination of the upper airway (LM and NP scores, SST‐12) and lower airway (spirometry, PEF recording and eNO).

Asthma is known to exacerbate the symptoms of CRSwNP. These results show that asthma and uncontrolled asthma are commonly found in patients with uncontrolled CRSwNP undergoing sinus surgery. These patients benefit from a comprehensive evaluation of both upper and lower airway diseases preoperatively.

## Author Contributions

Data collection was performed by E.G., J.S., A.L.‐H., M.L., S.H.‐M., L.H., A.M., S.V., S.T.‐S., and P.V. The planning of the study, data analyses, and interpretation were performed by E.G., P.K., S.T.‐S., and P.V. The manuscript was written by E.G., P.K., J.S., A.L.‐H., M.L., S.H.‐M., L.H., A.M., S.V., S.T.‐S., and P.V.

## Ethics Statement

Research permission (HUS/66/2018) was granted on June 21, 2018, by the Institutional Review Board of Helsinki University Hospital and ENT Hospital and was extended on February 22, 2023 (HUS/53/2023). The Research Ethical Board of the Helsinki and Uusimaa Hospital District approved the study design (HUS/1801/2017) on September 14, 2017. Informed consent was obtained from the study participants. The study was registered in ClinicalTrials.gov, NCT03704415.

## Conflicts of Interest

E.G. reports a lecture fee from GlaxoSmithKline, a manuscript fee from the Finnish doctors' society Duodecim, and an abroad congress fee from Orion. P.K. reports lecturing fees from GlaxoSmithKline, consultancies for Swedish Orphan Biovitrum and the Finnish Patient Insurance Center, grants from Allergy Research Foundation, positions as Principal Investigator for Theravance Pan Janus kinase inhibitor TD‐0903 and president of the Finnish Respiratory Society. J.S. reports a congress fee from Sanofi and consultancy for GlaxoSmithKline. A.L.‐H. reports consultancies for GlaxoSmithKline, Sanofi and Galenus Health, congress fees from Karl Storz, a confidential post at European Rhinologic Society delegate and a grant from Sanofi. S.T.S. reports consultancies for ALK‐Abelló, AstraZeneca, Clario, GlaxoSmithKline, Novartis, Sanofi, Orion, Roche Products and grants from GlaxoSmithKline and Sanofi. P.V. reports lecturing fees from Sanofi and GlaxoSmithKline. M.L., S.H.‐M., L.H., A.M. and S.V. report no conflicts of interest.

## Data Availability

The data are available from the corresponding author upon reasonable request. They are stored in a controlled access data storage at Helsinki University Hospital.
